# Comparison of perioperative outcomes with or without routine chest tube drainage after video-assisted thoracoscopic pulmonary resection: A systematic review and meta-analysis

**DOI:** 10.3389/fonc.2022.915020

**Published:** 2022-08-08

**Authors:** Rongyang Li, Jianhao Qiu, Chenghao Qu, Zheng Ma, Kun Wang, Yu Zhang, Weiming Yue, Hui Tian

**Affiliations:** Department of Thoracic Surgery, Qilu Hospital of Shandong University, Jinan, Shandong, China

**Keywords:** no routine chest tube drainage strategy, traditional chest tube drainage, video-assisted thoracoscopic lung resection, perioperative outcomes, systematic review, meta-analysis

## Abstract

**Background:**

In recent years, an increasing number of thoracic surgeons have attempted to apply no routine chest tube drainage (NT) strategy after thoracoscopic lung resection. However, the safety and feasibility of not routinely placing a chest tube after lung resection remain controversial. This study aimed to investigate the effect of NT strategy after thoracoscopic pulmonary resection on perioperative outcomes.

**Methods:**

A comprehensive literature search of PubMed, Embase, and the Cochrane Library databases until 3 January 2022 was performed to identify the studies that implemented NT strategy after thoracoscopic pulmonary resection. Perioperative outcomes were extracted by 2 reviewers independently and then synthesized using a random-effects model. Risk ratio (RR) and standardized mean difference (SMD) with 95% confidence interval (CI) served as the summary statistics for meta-analysis. Subgroup analysis and sensitivity analysis were subsequently performed.

**Results:**

A total of 12 studies with 1,381 patients were included. The meta-analysis indicated that patients in the NT group had a significantly reduced postoperative length of stay (LOS) (SMD = -0.91; 95% CI: -1.20 to -0.61; P < 0.001) and pain score on postoperative day (POD) 1 (SMD = -0.95; 95% CI: -1.54 to -0.36; P = 0.002), POD 2 (SMD = -0.37; 95% CI: -0.63 to -0.11; P = 0.005), and POD 3 (SMD = -0.39; 95% CI: -0.71 to -0.06; P = 0.02). Further subgroup analysis showed that the difference of postoperative LOS became statistically insignificant in the lobectomy or segmentectomy subgroup (SMD = -0.30; 95% CI: -0.91 to 0.32; P = 0.34). Although the risk of pneumothorax was significantly higher in the NT group (RR = 1.75; 95% CI: 1.14–2.68; P = 0.01), the reintervention rates were comparable between groups (RR = 1.04; 95% CI: 0.48–2.25; P = 0.92). No significant difference was found in pleural effusion, subcutaneous emphysema, operation time, pain score on POD 7, and wound healing satisfactory (all P > 0.05). The sensitivity analysis suggested that the results of the meta-analysis were stabilized.

**Conclusions:**

This meta-analysis suggested that NT strategy is safe and feasible for selected patients scheduled for video-assisted thoracoscopic pulmonary resection.

**Systematic Review Registration:**

https://inplasy.com/inplasy-2022-4-0026, identifier INPLASY202240026.

## Introduction

Lung cancer is the fastest-growing malignancy worldwide in morbidity and mortality, the most common cause of cancer death in men and the second leading cause of cancer death in women (1). Due to the popularization of low-dose computed tomography (CT) screening, the rate of detection of small pulmonary nodules (especially ground-glass nodules) has significantly increased in recent years, which makes early diagnosis and treatment of lung cancer more challenging (2, 3). With the rapid development of minimally invasive techniques, traditional thoracotomy has been transformed into video-assisted thoracoscopic surgery (VATS) with less risk to patients (4, 5). In general, a chest tube is routinely placed in the pleural cavity to mitigate against possible air leaks, hemorrhage, and chylothorax after VATS (6). However, some side effects of chest tube insertion are still difficult to avoid, such as increased postoperative pain and hindrance to postoperative activity, which could impede patient functional rehabilitation and significantly prolong postoperative length of stay (LOS) (7, 8).

Enhanced recovery after surgery (ERAS) is a multimodal perioperative management strategy first proposed by Dr. Engelman in 1994 in order to reduce postoperative pain, promote patients’ recovery, reduce the cost of hospitalization, and shorten the length of hospital stay (9). In recent years, this multidisciplinary perioperative rehabilitation concept has been widely applied in thoracic surgery with satisfactory results (10). An increasing number of thoracic surgeons, in order to promote the idea of ERAS, have attempted to apply no routine chest tube drainage (NT) strategy after thoracoscopic lung resection (11, 12). However, the increased incidence of postoperative pneumothorax and poor recruitment of the lungs are the main issues caused by the NT strategy (13).

Although several centers have conducted studies to explore the effect of NT strategy for thoracoscopic pulmonary resection in recent years, the safety and feasibility of not routinely placing a chest tube after lung resection remain controversial. A meta-analysis performed by Li et al. (14), including 6 retrospective and 3 prospective cohort studies, demonstrated that it was feasible and safe to omit chest tube after VATS for carefully selected patients. However, inappropriate inclusion criteria and relatively small sample sizes may introduce considerable bias, thereby reducing the reliability of the results. In addition, the perioperative outcomes that they reported were not comprehensive enough. To arrive at a more substantial conclusion, we aimed to conduct a systematic review and meta-analysis to determine the effect of NT strategy after thoracoscopic pulmonary resection on perioperative outcomes.

## Materials and methods

This systematic review and meta-analysis was reported in accordance with the Preferred Reporting Items for Systematic Reviews and Meta-Analyses (PRISMA) and the Meta-Analysis of Observational Studies in Epidemiology (MOOSE) guidelines and statement (15, 16). The protocol of this systematic review and meta-analysis has been registered on the INPLASY website (https://inplasy.com/inplasy-2022-4-0026); the registration number is INPLASY202240026.

### Databases and search strategy

The literature review was performed by relying on 3 online databases: PubMed, Embase, and the Cochrane Library until 3 January 2022. The Medical Subject Headings (MeSH) considered in the search strategy were “pulmonary neoplasms,” “thoracoscopic,” and “chest tube,” free of charge terms accessed in PubMed. Keywords and free words are used in each valid combination of the 2 Boolean operators (“AND” and “OR”). Search strategies for all databases are detailed in **Table S1**. Articles were individually evaluated and cross-checked by 2 authors (RL and JQ). In addition, we manually scanned the reference list of excluded publications to indicate any additional viable non-duplicate studies. Any differences between the reviewers are resolved through discussion.

### Study selection and criteria

The selection criteria were as follows: 1) involved adult patients who underwent selective thoracoscopic pulmonary resection (wedge resection, segmentectomy, and lobectomy); 2) involved a group that implemented NT strategy, including prophylactic air-extraction catheter insertion procedure (PC) or complete omission of chest tube drainage (OT); 3) involved a routine chest tube drainage (RT) group as control; 4) reported at least one of the relevant outcomes of interests (see below); 5) written in English.

The criteria for exclusion were as follows: 1) ineligible article types such as case reports, reviews, conference abstracts, non-comparative studies; 2) no results of interest existed; 3) non-human participants were included; 4) written in languages other than English.

### Endpoints and outcome measures

The primary outcome was postoperative LOS, which was defined as the time from surgery to recovery and discharge. Other related outcomes included operation duration, postoperative complications (including pneumothorax, pleural effusion, and subcutaneous emphysema), reintervention rates, postoperative pain scores, and wound healing satisfaction. Reintervention was defined as chest tube reinsertion or thoracentesis.

### Data collection

The 2 reviewers (RL and JQ) independently browsed eligible studies and extracted the corresponding data to fill in predefined forms. Any differences could be resolved by consensus. The following data were extracted from each study: 1) publication data: authors, published year, and country; 2) experimental data: study design and period, surgical procedure, and NT strategy; 3) demographic data: sample size, age, and gender; 4) outcome data: postoperative LOS, operation duration, postoperative complications in detail, postoperative pain score, postoperative reintervention rate, and wound healing satisfaction. We did not contact the authors for any unpublished data.

### Quality assessment

The quality of cohort studies was evaluated using the Newcastle–Ottawa Quality Assessment Scale (NOS) (17). We determined that studies with a score comparable to or higher than 6 were applicable to further meta-analysis. The Cochrane risk of bias tool was used to assess the quality of randomized controlled trials (RCTs) (18). Due to the nature of the interventions associated with the NT strategy, it is often not feasible to blind patients and staff. Therefore, if a study does not address blinding, a high risk of performance bias is assumed. The quality of each study was independently appraised by two investigators (RL and JQ). Any disagreement on quality assessment should be resolved by consensus.

### Statistical analysis

We calculated the risk ratio (RR) with 95% confidence interval (CI) to summarize the effects of NT strategy on dichotomous data. The standardized mean difference (SMD) with 95% CI appeared as the suitable statistics to summarize the mean values with standard deviations (SDs) for continuous variables. If the SDs were not provided, we would not incorporate the data in the quantitative synthesis because the extrapolation of SDs was only applicable for studies with a large sample size and normal distribution of outcomes due to the guidelines of the Cochrane Collaboration (18).

The Cochrane Q test and I^2^ statistics were used to quantify the heterogeneity level. An I^2^ greater than 50% is considered to have considerable heterogeneity (19). A 2-sided P value <0.05 was defined as statistical significance. In our study, random-effects models were applied to calculate pooled effect sizes in order to decrease possible bias. Egger’s test was used to detect any probable publication bias (20), and a significant publication bias was identified if Egger’s P < 0.05.

A sensitivity analysis was performed to further examine the stability of pooled estimates, in which the impact of each study on the overall estimates could be detected by omitting individual studies sequentially. In order to evaluate the effect of NT strategy on postoperative recovery for different surgical methods, a meta-analysis of postoperative LOS was then performed on 2 subgroups: wedge resection and segmentectomy or lobectomy.

All statistical analyses were conducted using the Review Manager software (RevMan version 5.3; The Nordic Cochrane Center, The Cochrane Collaboration, 2014) and Stata software (version 14.2; StataCorp LLC, College Station, TX, USA).

## Results

### Literature search

**Figure 1:**
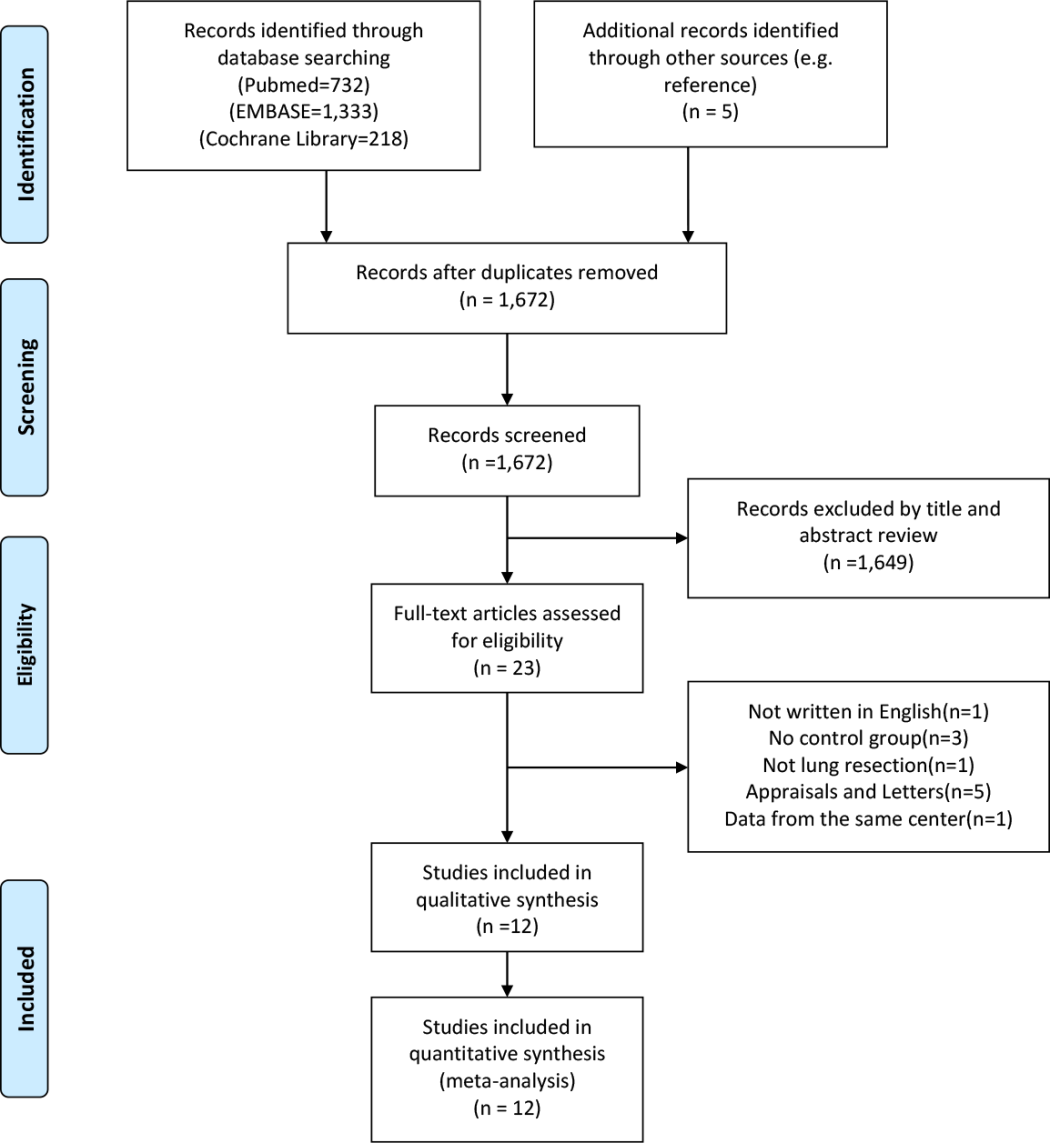
PRISMA flow diagram of literature retrieval. PRISMA, Preferred Reporting Items for Systematic Reviews and Meta-Analyses.

A flowchart outlining the search process was presented in **Figure 1**. A total of 2,283 potential articles were identified, including 732 PubMed citations, 1,333 Embase citations, and 218 Cochrane Library citations. In addition, manual searches of the literature in the reference list also yielded 5 relevant studies. After checking for duplicates and screening titles, abstracts, and full text, a total of 12 articles were finally included in our meta-analysis (21–32).

### Characteristics of the included studies

Baseline characteristics of each eligible study were summarized in **Table 1**, and the perioperative outcomes were presented in **Tables 2**–**4**. This meta-analysis involved 9 retrospective cohort studies (22–26, 28–30, 32), 1 prospective cohort study (27), and 2 RCTs (21, 31). The studies were conducted in 3 different countries during the period from 1998 to 2020, and the sample size varied from 50 to 333. A total of 1,381 patients eventually entered the meta-analysis, of which 764 patients were finally assigned to the NT group and 615 patients to the RT group. Approximately half of the participants were from China (n = 701; 50.8%), followed by 621 patients from Japan (45.0%) and 59 patients from the United States (4.3%). In terms of surgical methods, 1,169 cases underwent wedge pulmonary resection; the other 212 patients received segmentectomy or lobectomy.

**Table 1:** Baseline characteristics of the included studies.

Study (year)	Country	Study Period	Study type	No. of Patients			Age (years)		Gender (male ratio)		Surgical Procedure	NT Strategy
				Total	NT	RT	NT	RT	NT	RT		
Zhang et al., 2020 (31)	China	2017-2018	RCT	84	40	44	53.7 ± 11.5	54.4 ± 11.7	15 (37.5)	16 (36.4)	Wedge resection	PC
Liu Z et al., 2020 (23)	China	2018-2019	RCS	110	55	55	44.8 ± 11.1	45.1 ± 10.5	23 (41.8)	24 (43.6)	Wedge resection	OT
Liu C et al., 2020 (22)	China	2016-2019	RCS	135	122	13	47.8 ± 20.1	46.3 ± 19.1	65 (55.3)	12 (92.3)	Wedge resection	OT
Liao et al., 2019 (21)	China	2016-2017	RCT	100	50	50	52.4 ± 10.9	54.9 ± 10.1	6 (12.0)	7 (14.0)	Wedge resection	OT
Zhang et al., 2018 (32)	China	2015-2017	RCS	123	87	36	54 (28–81)	52 (16–82)	40 (46.0)	12 (33.3)	Wedge resection	PC/OT
Murakami et al., 2017 (25)	Japan	2012-2014	RCS	162	102	60	69.4 ± 10.6	71.3 ± 9.9	49 (47.1)	39 (65.0)	Lobectomy; Segmentectomy	OT
Lu et al., 2017 (24)	China	2013-2015	RCS	89	44	45	54.1 ± 12.9	57.0 ± 16.3	21 (47.7)	21 (46.7)	Wedge resection	OT
Yang et al., 2016 (30)	China	2015-2016	RCS	60	30	30	55.5 ± 8.4	59.4 ± 12.3	23 (76.7)	23 (76.7)	Wedge resection	OT
Ueda et al., 2013 (28)	Japan	2011-2012	RCS	50	29	21	71.7 ± 9.6	72.4 ± 11.2	13 (44.8)	16 (76.2)	Lobectomy; segmentectomy	OT
Nakashima et al., 2011 (26)	Japan	2000-2009	RCS	333	132	201	56 ± 15	55 ± 19	67 (50.8)	137 (68.2)	Wedge resection	OT
Watanabe et al., 2004 (29)	Japan	1998-2002	RCS	76	42	34	55 ± 15	53 ± 17	20 (47.6)	21 (61.8)	Wedge resection	OT
Russo et al., 1998 (27)	USA	1995-1997	PCS	59	31	26	61 (24–82)	62 (26–76)	16 (48.5)	13 (50.0)	Wedge resection	OT

NT, no routine chest tube drainage; RT, routine chest tube drainage; PC, prophylactic air-extraction catheter insertion procedure; OT, complete omission of chest tube drainage; USA, The United States of America; RCT, randomized controlled trial; RCS, retrospective cohort study; PCS, prospective cohort study; NR, not reported.

**Table 2:** Perioperative outcomes of the included studies.

Study (year)	Postoperative LOS (d)		Operation duration (min)		Reintervention (%)		Overall postoperative complications (%)		Wound healing satisfaction (%)	
	NT	RT	NT	RT	NT	RT	NT	RT	NT	RT
Zhang et al., 2020 (31)	2.5 ± 1.5	3.2 ± 2.1	60 (50–80)	60 (50–88)	0	1 (2.3)	NR	NR	38 (95.0)	38 (86.4)
Liu Z et al., 2020 (23)	1.5 ± 0.5	2.5 ± 0.8	59.3 ± 10.6	52.8 ± 11.4	1 (1.8)	1 (1.8)	NR	NR	54 (98.2)	51 (92.7)
Liu C et al., 2020 (22)	2.2 ± 0.9	4.1 ± 2.7	54.2 ± 19.5	53.8 ± 19.1	3 (2.5)	0	NR	NR	NR	NR
Liao et al., 2019 (21)	1.2 ± 0.5	2.6 ± 0.9	59.0 ± 15.8	73.7 ± 26.6	2 (4.0)	0	NR	NR	42 (84.0)	48 (96.0)
Zhang et al., 2018 (32)	3.1 ± 1.5	4.8 ± 2.9	74.6 ± 23.9	66.5 ± 27.5	4 (4.6)	1 (2.8)	NR	NR	NR	NR
Murakami et al., 2017 (25)	9.7 ± 3.8	12.9 ± 7.8	NR	NR	1 (1.0)	2 (3.3)	8 (7.8)	16 (26.7)	NR	NR
Lu et al., 2017 (24)	3.1 ± 1.0	4.1 ± 0.9	NR	NR	NR	NR	15 (34.1)	24 (53.3)	NR	NR
Yang et al., 2016 (30)	3.1 ± 0.7	4.4 ± 1.3	72.0 ± 21.3	79.1 ± 32.2	0	0	NR	NR	27 (90.0)	22 (73.3)
Ueda et al., 2013 (28)	13.3 ± 15.5	12.5 ± 6.6	152.0 ± 53.0	198.0 ± 78.0	0	1 (4.8)	NR	NR	NR	NR
Nakashima et al., 2011 (26)	4.6 ± 2.2	6.7 ± 4.4	NR	NR	4 (3.0)	3 (1.5)	11 (8.3)	10 (5.0)	NR	NR
Watanabe et al., 2004 (29)	3.2 ± 1.0	3.6 ± 1.5	NR	NR	2 (4.8)	2 (5.9)	4 (9.5)	2 (5.9)	NR	NR
Russo et al., 1998 (27)	2.0 ± 1.0	3.9 ± 2.1	NR	NR	0	0	NR	NR	NR	NR

NT, no routine chest tube drainage; RT, routine chest tube drainage; LOS, length of stay; NR, not reported.

**Table 3:** Detailed postoperative complications of the included studies.

Study (year)	Pneumothorax (%)		Pleural effusion (%)		Subcutaneous emphysema (%)		Pneumonia (%)		Arrhythmia (%)	
	NT	RT	NT	RT	NT	RT	NT	RT	NT	RT
Zhang et al., 2020 (31)	4 (10.0)	4 (9.1)	2 (5.0)	2 (4.5)	8 (20.0)	8 (18.2)	0	1 (2.3)	NR	NR
Liu Z et al., 2020 (23)	15 (27.3)	12 (21.8)	1 (1.8)	1 (1.8)	9 (16.4)	8 (14.6)	NR	NR	0	0
Liu C et al., 2020 (22)	35 (28.7)	0	16 (13.1)	0	17 (13.9)	0	NR	NR	NR	NR
Liao et al., 2019 (21)	18 (36.0)	5 (10.0)	4 (8.0)	3 (6.0)	11 (22.0)	6 (12.0)	NR	NR	NR	NR
Zhang et al., 2018 (32)	12 (13.8)	2 (5.6)	1 (1.1)	0	NR	NR	0	1 (2.8)	NR	NR
Murakami et al., 2017 (25)	NR	NR	NR	NR	NR	NR	3 (2.9)	5 (8.3)	5 (4.9)	4 (6.7)
Lu et al., 2017 (24)	0	0	NR	NR	15 (34.1)	24 (53.3)	NR	NR	NR	NR
Yang et al., 2016 (30)	12 (40.0)	4 (13.3)	1 (3.3)	0	2 (6.6)	0	NR	NR	NR	NR
Ueda et al., 2013 (28)	NR	NR	NR	NR	0	1 (4.8)	NR	NR	NR	NR
Nakashima et al., 2011 (26)	10 (7.6%)	8 (4.0)	1 (0.8)	1 (0.5)	NR	NR	NR	NR	NR	NR
Watanabe et al., 2004 (29)	4 (9.5)	2 (5.9)	0	0	NR	NR	NR	NR	NR	NR
Russo et al., 1998 (27)	5 (16.1)	7 (26.9)	0	0	NR	NR	NR	NR	NR	NR

NT, no routine chest tube drainage; RT, routine chest tube drainage; NR, not reported.

**Table 4:** Postoperative pain score of the included studies.

Study (year)	POD 1		POD 2		POD 3		POD 7		Pain scale
	NT	RT	NT	RT	NT	RT	NT	RT	
Zhang et al., 2020 (31)	1.6 ± 0.7	2.4 ± 1.2	NR	NR	NR	NR	NR	NR	NRS
Liu Z et al., 2020 (23)	1.0 ± 0.7	3.0 ± 0.9	NR	NR	0.5 ± 0.5	1.1 ± 1.5	0.4 ± 0.4	0.7 ± 0.4	VAS
Liu C et al., 2020 (22)	NR	NR	NR	NR	NR	NR	NR	NR	NR
Liao et al., 2019 (21)	0.9 ± 0.7	1.3 ± 0.9	0.5 ± 0.6	0.9 ± 1.6	NR	NR	NR	NR	VAS
Zhang et al., 2018 (32)	2.3 ± 0.9	3.4 ± 1.1	NR	NR	NR	NR	NR	NR	NRS
Murakami et al., 2017 (25)	NR	NR	NR	NR	NR	NR	NR	NR	NR
Lu et al., 2017 (24)	NR	NR	NR	NR	NR	NR	NR	NR	NR
Yang et al., 2016 (30)	1.0 ± 0.8	1.5 ± 1.1	0.6 ± 0.5	0.9 ± 0.5	NR	NR	NR	NR	VAS
Ueda et al., 2013 (28)	NR	NR	NR	NR	NR	NR	NR	NR	NR
Nakashima et al., 2011 (26)	NR	NR	NR	NR	NR	NR	NR	NR	NR
Watanabe et al., 2004 (29)	1.56 ± 0.42	1.71 ± 0.53	1.40 ± 0.40	1.51 ± 0.43	1.17 ± 0.43	1.25 ± 0.37	0.56 ± 0.31	0.60 ± 0.37	VAS
Russo et al., 1998 (27)	NR	NR	NR	NR	NR	NR	NR	NR	NR

NT, no routine chest tube drainage; RT, routine chest tube drainage; POD, postoperative day; VAS, visual analog scale; NRS, Numeric Rating Scale; NR, not reported.

### Quality assessment

The quality assessment of the included RCTs and cohort studies was presented in **Figure S1** and **Table S2**, respectively. The NOS scores of the 10 included cohort studies were all greater than 6, suggesting that they were all of acceptable quality. As for the other 2 included RCTs, all of them presented a high risk of performance and detection bias due to the nature of the interventions associated with the NT strategy. No other risk of bias was found.

### Postoperative length of stay

**Figure 2:**
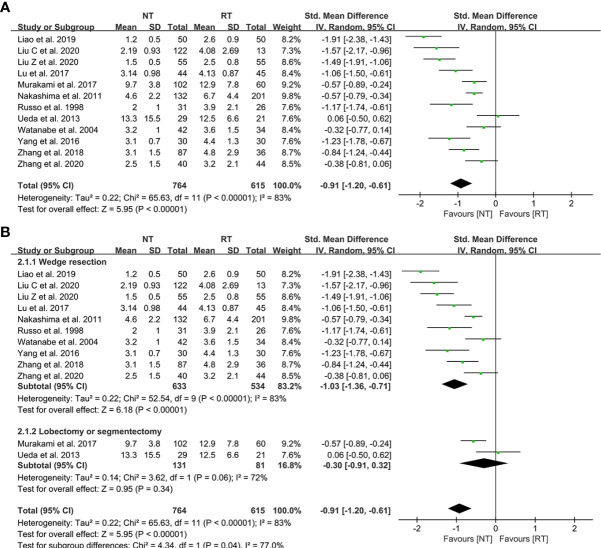
Meta-analysis and subgroup analysis of postoperative length of stay (LOS) between the NT and RT groups. **(A)** Meta-analysis of postoperative LOS; **(B)** Subgroup analysis of postoperative LOS. NT, no routine chest tube drainage; RT, routine chest tube drainage; CI, confidence interval.

All of the 12 eligible studies investigated the effect of NT strategy on the length of postoperative hospital stay. The meta-analysis indicated that postoperative LOS was shorter in the NT group (SMD = -0.91; 95% CI: -1.20 to -0.61; P < 0.001) with a considerable heterogeneity (I^2^ = 83%; P < 0.001), as shown in **Figure 2A**. No publication bias was found using Egger’s test (P = 0.196).

Further subgroup analysis were performed to evaluate the effect of NT strategy on postoperative recovery for different surgical methods. According to the different surgical procedures, the patients were divided into two subgroups: wedge resection group and segmentectomy or lobectomy group. As shown in **Figure 2B**, the postoperative LOS of the NT group was shorter than that of the RT group (SMD = -1.03; 95% CI: -1.36 to -0.71; P < 0.001) in the subgroup of wedge resection, while the postoperative LOS became comparable between the two groups in the segmentectomy or lobectomy subgroup (SMD = -0.30; 95% CI: -0.91 to 0.32; P = 0.34).

### Postoperative complications

**Figure 3:**
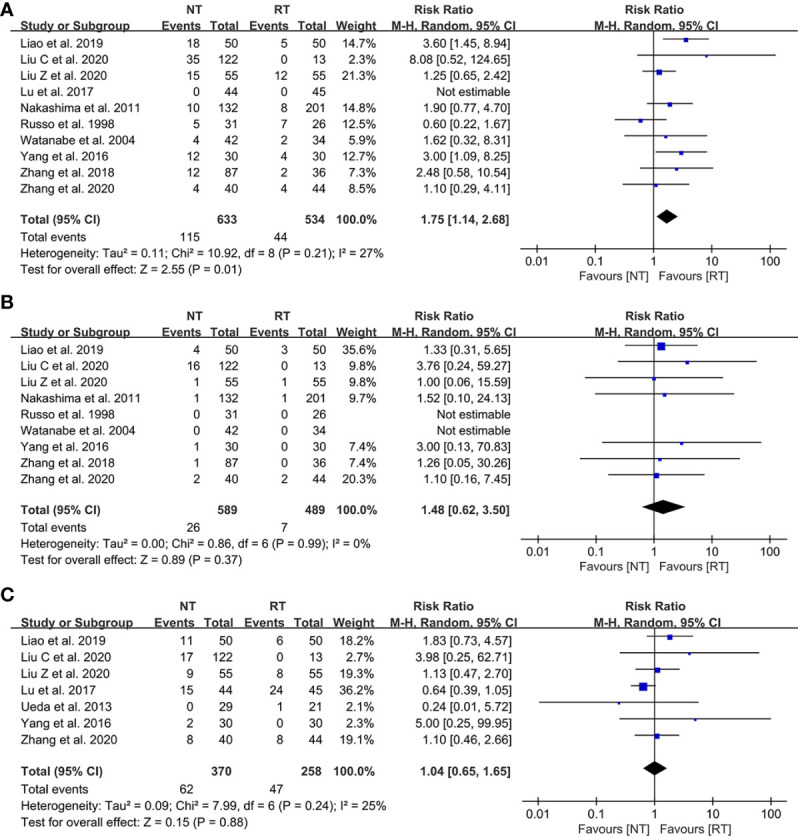
Meta-analysis of postoperative complications between the NT and RT groups. **(A)** Pneumothorax; **(B)** Pleural effusion; **(C)** Subcutaneous emphysema. NT, no routine chest tube drainage; RT, routine chest tube drainage; CI, confidence interval.

The detailed data on postoperative complications of the 12 eligible literatures were presented in **Table 3**. The meta-analysis indicated that the risk of postoperative pneumothorax was significantly higher in the NT group than that in the RT group (RR = 1.75; 95% CI: 1.14–2.68; P = 0.01) with a relatively low heterogeneity (I^2^ = 27%; P = 0.21), as shown in **Figure 3A**. No publication bias was found using Egger’s test (P = 0.450).

In contrast, there was no statistically significant difference in terms of the risks of postoperative pleural effusion (RR = 1.48; 95% CI: 0.62–3.50; P = 0.37; I^2^ = 0%) and subcutaneous emphysema (RR = 1.04; 95% CI: 0.65–1.65; P = 0.88; I^2^ = 25%) between the NT and RT groups with a slight heterogeneity, as shown in **Figures 3B, C**, respectively. No publication bias was found using Egger’s test (P = 0.335 for pleural effusion; P = 0.215 for subcutaneous emphysema).

### Reintervention

**Figure 4:**
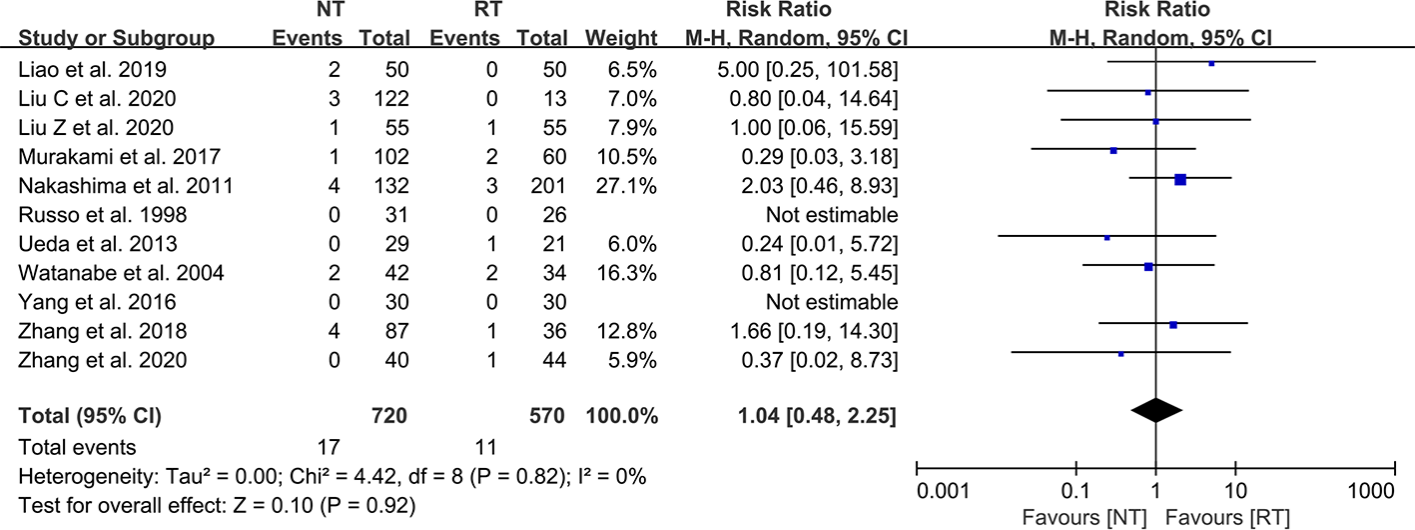
Meta-analysis of reintervention rate between the NT and RT groups. NT, no routine chest tube drainage; RT, routine chest tube drainage; CI, confidence interval.

A total of 11 included studies reported the incidence of postoperative reintervention for patients (21–23, 25–32). The results of the meta-analysis indicated that there was no significant difference in the postoperative reintervention rate between the NT group and the RT group (RR = 1.04; 95% CI: 0.48–2.25; *P* = 0.92) without heterogeneity (I^2^ = 0%; P = 0.82) (**Figure 4**). No publication bias was found using Egger’s test (P = 0.241).

### Postoperative pain score

**Figure 5:**
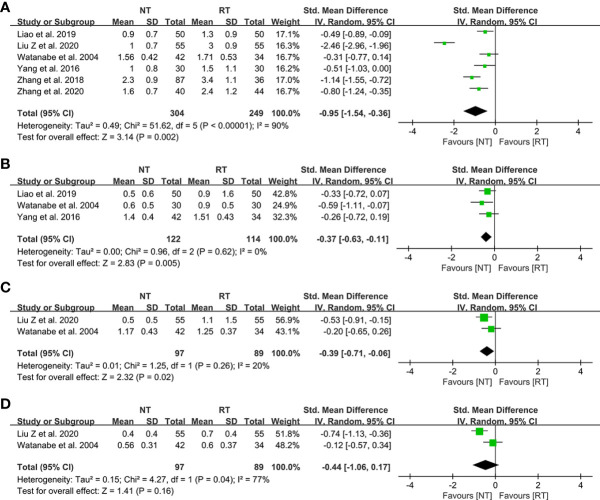
Meta-analysis of postoperative pain score between the NT and RT groups. **(A)** POD 1; **(B)** POD 2; **(C)** POD 3; **(D)** POD 7. NT, no routine chest tube drainage; RT, routine chest tube drainage; POD, postoperative day; CI, confidence interval.

The detailed data on postoperative pain score were presented in **Table 4**. As shown in **Figures 5A–C**, patients in the NT group experienced a lower pain score on POD 1 (SMD = -0.95; 95% CI: -1.54 to -0.36; P = 0.002), POD 2 (SMD = -0.37; 95% CI: -0.63 to -0.11; P = 0.005), and POD 3 (SMD = -0.39; 95% CI: -0.71 to -0.06; P = 0.02) compared with the RT group. However, the meta-analysis indicated that the pain scores of patients on POD 7 became comparable between the two groups (SMD = -0.44; 95% CI, -1.06 to 0.17; P = 0.16) (**Figure 5D**).

### Operation duration

**Figure 6:**
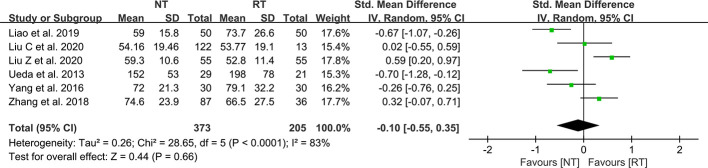
Meta-analysis of operation duration between the NT and RT groups. NT, no routine chest tube drainage; RT, routine chest tube drainage; CI, confidence interval.

As shown in **Table 2**, seven studies mentioned the duration of surgery, of which 6 studies present the data as mean ± SD (21–23, 28, 30–32). The results suggested that there was no statistical difference between the NT group and the RT group in terms of operative duration (SMD = -0.10; 95% CI: -0.55 to 0.35; P = 0.66) with a considerable heterogeneity (I^2^ = 83%; P < 0.01) (**Figure 6**). No publication bias was found using Egger’s test (P = 0.351).

### Wound healing satisfaction

**Figure 7:**
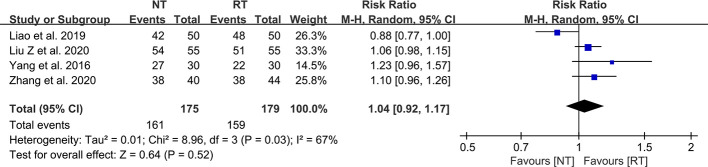
Meta-analysis of wound healing satisfaction between the NT and RT groups. NT, no routine chest tube drainage; RT, routine chest tube drainage; CI, confidence interval.

As demonstrated in **Figure 7**, there was no statistically significant difference in the wound healing satisfaction between the NT and RT groups (RR = 1.04; 95% CI: 0.92–1.17; P = 0.52) with high heterogeneity (I^2^ = 67%; P = 0.03).

### Sensitivity analysis

We performed the sensitivity analysis by omitting individual studies sequentially. None of the summary RRs based on the remaining studies in each component analysis exceeded the estimated range, as shown in **Figure S2**. Nor was there any substantial change between the adjusted pooled estimates and the major aggregate estimates. The robustness of our meta-analysis was thus confirmed.

## Discussion

The placement of routine chest tube drainage after thoracoscopic pulmonary resection has already been the gold standard approach to prevent postoperative pneumothorax and pleural effusion (33). In recent years, an increasing number of thoracic surgeons, in order to realize the concept of ERAS, have attempted to not routinely place chest tube drainage and instead use the method of prophylactic air-extraction catheter insertion procedure or complete omission of chest tube drainage (21, 32). However, the safety and feasibility of not routinely placing a chest tube after lung resection remain controversial. To date, no meta-analysis has been conducted to comprehensively compare the perioperative outcomes between with and without routine chest tube drainage after video-assisted thoracoscopic pulmonary resection. Therefore, we performed a systematic review and meta-analysis including 12 comparative studies on this subject to further identify the safety and feasibility of the NT strategy.

In this study, we found that the no routine placement of chest tube drainage after thoracoscopic lung resection can significantly shorten the postoperative hospital stay (SMD = -0.91; 95% CI: -1.20 to -0.61; P < 0.001). However, the meta-analysis of postoperative LOS showed a relatively high heterogeneity (I^2^ = 83%; P < 0.001), which might derive from the different surgical approaches (wedge resection, segmentectomy, and lobectomy) and medical insurance of regions and countries. For example, the healthcare system in Japan allows patients to stay in the hospital for a relatively long period of time, even if they have already met discharge criteria (34). It is worth mentioning that the difference of postoperative LOS became statistically insignificant in the lobectomy or segmentectomy subgroup (SMD = -0.30; 95% CI: -0.91 to 0.32; P = 0.34). However, this might be caused by too few (only two) studies in this subgroup, and the effectiveness of NT strategy for lobectomy or segmentectomy warrants further exploration in future studies.

The main concerns caused by omitting routine placement of chest tube drainage after pulmonary resection are the risks of pneumothorax, bleeding, pleural effusion, and subcutaneous emphysema (8, 35). In terms of postoperative complications, we did not perform a pooled analysis of the overall incidence of complications because of the small number of studies reporting it. Instead, we performed a meta-analysis of more detailed complications. The results of the meta-analysis indicated that the incidence of pneumothorax was significantly increased in the NT group (RR = 1.75; 95% CI: 1.14–2.68; P = 0.01). However, there was no significant difference between the two groups in the incidence of pleural effusion (RR = 1.48; 95% CI: 0.62–3.50; P = 0.37) and subcutaneous emphysema (RR = 1.04; 95% CI: 0.65–1.65; P = 0.88). Notably, the reintervention rates of the NT group did not significantly increase (RR = 1.04; 95% CI: 0.48–2.25; P = 0.92), suggesting that the vast majority of pneumothorax could be self-absorbed safely without chest tube reinsertion or thoracocentesis.

The traditional drainage tube is often reported as one of the main reasons of postoperative pain and might interfere with postoperative activity, which could prevent patients from functional rehabilitation and thus prolong the duration of hospitalization (21, 36). In this study, we found that the pain scores on POD 1 (SMD = -0.95; 95% CI: -1.54 to -0.36; P = 0.002), POD 2 (SMD = -0.37; 95% CI: -0.63 to -0.11; P = 0.005), and POD 3 (SMD = -0.39; 95% CI: -0.71 to -0.06; P = 0.02) were significantly decreased without routine chest tube placement. However, the pain scores became comparable between the two groups on POD 7 (SMD = -0.44; 95% CI: -1.06 to 0.17; P = 0.16), indicating that the chest tube is one of the major sources of postoperative pain. Enhanced postoperative pain would prevent patients from effective coughing and thus deteriorate the ventilation capacity. A study performed by Ueda et al. (37) in 2019 showed that the omission of chest tube drainage could reduce the pain and preserve the ventilatory capacity and exercise capacity in the early postoperative period for patients undergoing thoracoscopic pulmonary resection. In addition, there was no significant different in wound healing satisfaction postoperatively between the two groups (RR = 1.04; 95% CI: 0.92–1.17; P = 0.52), which might be attributed to the benefits of minimally invasive technology such as video-assisted thoracoscopic surgery.

To ensure the security of the NT strategy, patients should undergo rigorous air tightness tests before being assigned to the NT group. Water-seal air tightness test and suction-induced air leakage test are relatively common methods to test air leaks during the operation and were applied in majority of the studies. If no air leaks were observed in the air tightness tests, then the patients would be assigned to the NT group. Liu Z et al. (23) have reported a modified air leak test in 2020. The water-seal test was first used at the end of the operation, and then patients were changed to reverse Trendelenburg position with 30° with a chest tube placed at the posterior one-third position of the incision to further test for existence of air leaks. They suggested that complete air drainage is more easily achieved by a chest tube in this position (23). In recent years, a digital drainage system (DDS) has also been used for air tightness tests. A single chest tube was placed through the incision into the pleural cavity before closing the incision and was connected to a DDS. If the DDS indicated 0 ml/min airflow before completion of the wound closure, the chest tube would be removed immediately (22, 38). A study performed by Russo et al. (27) in 1998 used an early removal of chest tube approach. Patients assigned to the NT group had their chest tubes removed within 90 min postoperatively in the recovery room (27). Although this approach was not a strict NT strategy, we still included this study in our analysis because traditional chest tube management tends to keep the chest tube inserted for at least 24 h. Some argued that the operation duration may be extended due to the implementation of the air tightness tests (23, 32). However, our meta-analysis suggested that the operation duration was comparable between the two groups (SMD = -0.10; 95% CI: -0.55 to 0.35; P = 0.66).

At present, the NT strategy mainly includes two methods: prophylactic air-extraction catheter insertion procedure and complete omission of chest tube drainage. Prophylactic air-extraction catheter insertion procedure was first reported by Zhang et al. (32) in 2018. In this procedure, a two-lumen central venous catheter (20 cm, 7 Fr) was inserted into the second intercostal space before directly closing the incision. The air extraction was performed using an injector through the preset catheter if the chest roentgenogram revealed a pneumothorax on POD 1. A recent randomized clinical trial performed by Zhang et al. (31) in 2020 has demonstrated that the prophylactic air-extraction catheter insertion was a safe procedure that could reduce pain and facilitate patients’ recovery after pulmonary wedge resection. However, which of the two methods is better has not been discussed. We originally intended to conduct a subgroup analysis to explore this issue, but due to the insufficient data on prophylactic air-extraction catheter procedure, our idea was not implemented, which could be considered in a future meta-analysis.

It is noteworthy that the selection criteria for patients who do not routinely place chest tubes after video-assisted thoracoscopic pulmonary resection are relatively strict. Important factors that should be considered when selecting patients are the following: 1) absence of air leaks during the intraoperative air tightness tests, 2) absence of dense pleural adhesion, 3) absence of a history of previous ipsilateral thoracic surgery, 4) absence of moderate-to-severe obstructive or restrictive pulmonary diseases.

This study has several limitations that should be considered. First, the majority of the included studies were single-center retrospective cohort studies, and only 2 RCTs were included. Some biases common to cohort studies are unavoidable, such as cohort selection bias, which might have reduced the reliability of the results. Second, different surgical approaches and different pain rating scales were included in this meta-analysis, which inevitably increase the clinical heterogeneity. In addition, prophylactic air-extraction catheter insertion procedure and complete omission of chest tube drainage were both included in the NT group, possibly leading to heterogeneity of the results. Third, although 12 studies were included for analysis, not all studies reported the outcomes we were interested in and we just used the available data to analyze in each comparison. In addition, we did not perform subgroup analyses for outcomes other than postoperative LOS due to the limited data reported. Fourth, all of the studies included had their own criteria to select patients into the NT groups; this might lead to different baseline characteristics of the two groups and a high clinical heterogeneity. Finally, a certain language-based bias might have arisen due to the requirement of full-text English language literature.

## Conclusion

This systematic review and meta-analysis is the most up-to-date and comprehensive review of the literature on the NT strategy after video-assisted thoracoscopic pulmonary resection. The NT strategy could not only significantly shorten the postoperative LOS but also reduce short-term postoperative pain for patients without increasing the reintervention rate, suggesting that it is safe and feasible for selected patients scheduled for video-assisted thoracoscopic pulmonary resection.

## Data availability statement

The original contributions presented in the study are included in the article/**Supplementary Material**. Further inquiries can be directed to the corresponding author.

## Author contributions

Conception and design: RL and HT. Administrative support: HT, WY and ZM. Provision of study materials or patients: RL and JQ. Collection and assembly of data: RL, JQ, KW and YZ. Data analysis and interpretation: RL, JQ and CQ. All authors contributed to the article and approved the submitted version.

## Funding

This work was funded by National Key Research and Development Program (2021YFC2500904, and 2021YFC2500905), Natural Science Foundation of Shandong Province (ZR2021LSW006) and the Taishan Scholar Program of Shandong Province (no. ts201712087).

## Conflict of interest

The authors declare that the research was conducted in the absence of any commercial or financial relationships that could be construed as a potential conflict of interest.

## Publisher’s note

All claims expressed in this article are solely those of the authors and do not necessarily represent those of their affiliated organizations, or those of the publisher, the editors and the reviewers. Any product that may be evaluated in this article, or claim that may be made by its manufacturer, is not guaranteed or endorsed by the publisher.

## Supplementary material

Supplementary Figure 1 Detailed quality assessment of included RCTs. **(A)** Risk of bias summary; **(B)** Risk of bias graph.

Supplementary Figure 2 Sensitivity analyses of outcomes. **(A)** Postoperative LOS; **(B)** Pneumothorax; **(C)** Pleural effusion; **(D)** Subcutaneous emphysema; **(E)** Reintervention rate; **(F)** Pain score on POD 1; **(G)** Operation duration; **(H)** Wound healing satisfaction. CI, confidence interval.
