# Supplementary figures and images for: Comparison of perioperative outcomes with or without routine chest tube drainage after video-assisted thoracoscopic pulmonary resection: A systematic review and meta-analysis
Source: Front Oncol. 2022 Aug 8;12:915020. doi: 10.3389/fonc.2022.915020 (PMC9393739; doi:10.3389/fonc.2022.915020)

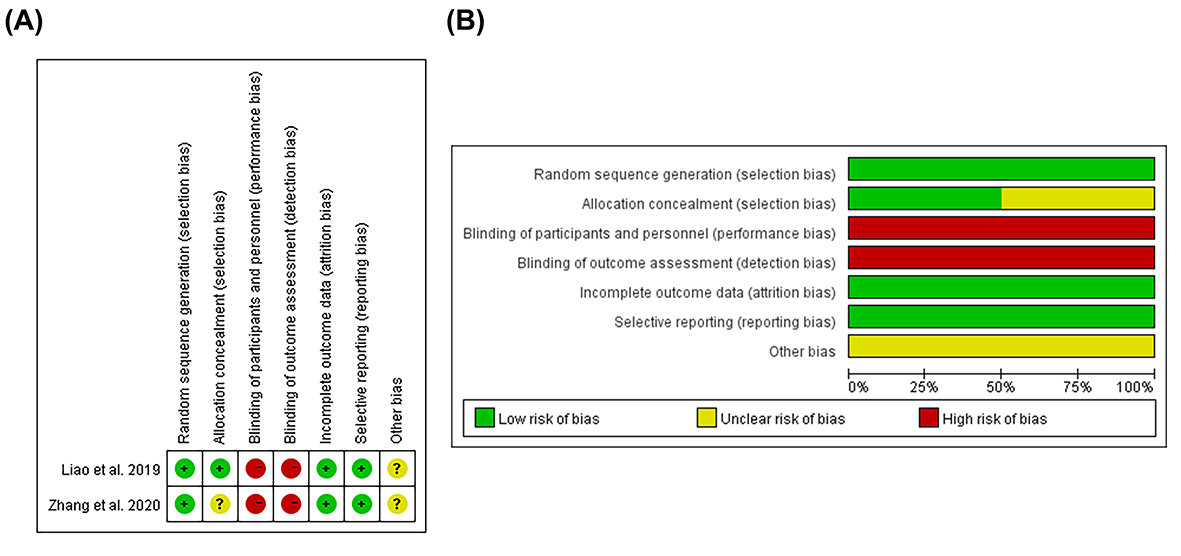

Supplement: Supplementary Figure 1 — Detailed quality assessment of included RCTs. (A) Risk of bias summary; (B) Risk of bias graph. [file Image_1.tif]

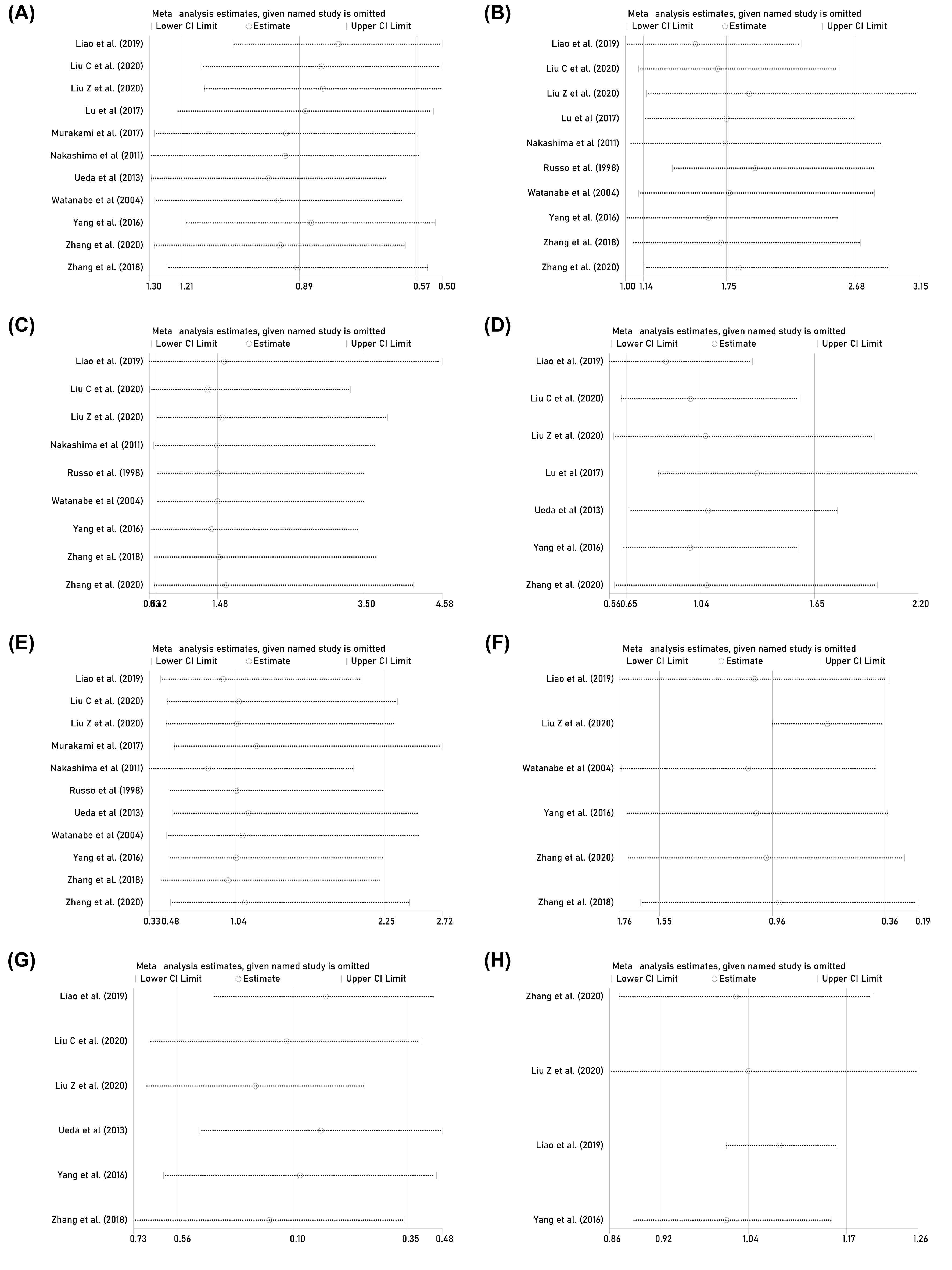

Supplement: Supplementary Figure 2 — Sensitivity analyses of outcomes. (A) Postoperative LOS; (B) Pneumothorax; (C) Pleural effusion; (D) Subcutaneous emphysema; (E) Reintervention rate; (F) Pain score on POD 1; (G) Operation duration; (H) Wound healing satisfaction. CI, confidence interval. [file Image_2.tif]
