# Supplementary material for: Comparison of perioperative outcomes with or without routine chest tube drainage after video-assisted thoracoscopic pulmonary resection: A systematic review and meta-analysis
Source: Front Oncol. 2022 Aug 8;12:915020. doi: 10.3389/fonc.2022.915020 (PMC9393739; doi:10.3389/fonc.2022.915020)
Supplement: Supplementary file 3 [file Table_1.docx]

| **Database** | **Search Strategy** |
| --- | --- |
| Pubmed | ((((((((((((((((((("Lung Neoplasms"[Mesh]) OR (Pulmonary Neoplasms[Title/Abstract])) OR (Neoplasms, Lung[Title/Abstract])) OR (Lung Neoplasm[Title/Abstract])) OR (Neoplasm, Lung[Title/Abstract])) OR (Neoplasms, Pulmonary[Title/Abstract])) OR (Neoplasm, Pulmonary[Title/Abstract])) OR (Pulmonary Neoplasm[Title/Abstract])) OR (Lung Cancer[Title/Abstract])) OR (Cancer, Lung[Title/Abstract])) OR (Cancers, Lung[Title/Abstract])) OR (Lung Cancers[Title/Abstract])) OR (Pulmonary Cancer[Title/Abstract])) OR (Cancer, Pulmonary[Title/Abstract])) OR (Cancers, Pulmonary[Title/Abstract])) OR (Pulmonary Cancers[Title/Abstract])) OR (Cancer of the Lung[Title/Abstract])) OR (Cancer of Lung[Title/Abstract])) AND ((((((((("Thoracoscopy"[Mesh]) OR (Thoracoscopic[Title/Abstract])) OR (Thoracic Surgery Video-Assisted[Title/Abstract])) OR (Thoracic Surgery Robotic-Assisted[Title/Abstract])) OR (Video-Assisted Thoracic Surgery[Title/Abstract])) OR (Robot-Assisted Thoracic Surgery[Title/Abstract])) OR (VATS[Title/Abstract])) OR (Lung Resection[Title/Abstract])) OR (Pulmonary resection[Title/Abstract]))) AND ((((((("Drainage"[Mesh]) OR (Drainage Tube[Title/Abstract])) OR (Chest Tube[Title/Abstract])) OR (Chest Drain[Title/Abstract])) OR (Chest Drainage[Title/Abstract])) OR (Nonintubated[Title/Abstract])) OR (Tubeless[Title/Abstract])) |
| Embase | ('cancer of lung' OR 'cancer of the lung' OR 'pulmonary cancers' OR 'pulmonary cancer' OR 'lung cancer' OR 'lung cancers' OR 'pulmonary neoplasm' OR 'lung neoplasm' OR 'pulmonary neoplasms':ab,ti OR 'lung cancer'/exp) AND (thoracoscopic OR 'thoracic surgery video-assisted' OR 'thoracic surgery robotic-assisted' OR 'video-assisted thoracic surgery' OR 'robot-assisted thoracic surgery' OR vats OR 'lung resection' OR 'pulmonary resection':ab,ti OR 'thoracoscopy'/exp) AND ('drainage tube' OR 'chest tube' OR 'chest drain' OR 'chest drainage' OR nonintubated OR tubeless:ab,ti OR 'drainage'/exp) |
| Cochrane Library | ((LUNG NEOPLASM[MeSH]) OR (((LUNG OR PULMON*) AND (NEOPLAS* OR CANCER OR CARCINOMA*)):ti,ab,kw) AND (((Drainage Tube OR Chest Tube OR Chest Drain OR Chest Drainage OR Nonintubat* OR Tubeless):ti,ab,kw) OR (Drainage[MeSH])) AND (((Thoracic Surgery Video-Assisted OR Thoracic Surgery Robotic-Assisted OR Video-Assisted Thoracic Surgery OR Robot-Assisted Thoracic Surgery OR VATS OR Lung resection OR Pulmonary resection OR Thoracoscop*):ti,ab,kw) OR (Thoracoscopy[MeSH])) |

**Table S1.** Details of search strategy for all databases
