# Supplementary material for: Comparison of perioperative outcomes with or without routine chest tube drainage after video-assisted thoracoscopic pulmonary resection: A systematic review and meta-analysis
Source: Front Oncol. 2022 Aug 8;12:915020. doi: 10.3389/fonc.2022.915020 (PMC9393739; doi:10.3389/fonc.2022.915020)
Supplement: Supplementary file 4 [file Table_2.docx]

| **Items of NOS** | **Included Studies** | | | | | | | | | |
| --- | --- | --- | --- | --- | --- | --- | --- | --- | --- | --- |
|  | Liu Z *et al.* 2020 | Liu C *et al.* 2020 | Zhang *et al.* 2018 | Murakami *et al.* 2017 | Lu *et al.* 2017 | Yang *et al.* 2016 | Ueda *et al.* 2013 | Nakashima *et al.* 2011 | Watanabe *et al.* 2004 | Russo *et al.* 1998 |
| **Selection** |  |  |  |  |  |  |  |  |  |  |
| Representativeness of the exposed cohort | **★** | **★** | **★** | **★** | **★** | **★** | **★** | **★** | **★** | **★** |
| Selection of the non-exposed cohort | **★** |  | **★** |  | **★** | **★** |  | **★** | **★** | **★** |
| Ascertainment of exposure | **★** | **★** | **★** | **★** | **★** | **★** | **★** | **★** | **★** | **★** |
| Demonstration that outcome of interest was not present at start of study | **★** | **★** | **★** | **★** | **★** | **★** | **★** | **★** | **★** | **★** |
| **Comparability** |  |  |  |  |  |  |  |  |  |  |
| Comparability of cohorts on basis of the design or analysis | **★★** | **★** | **★★** | **★** | **★** | **★★** | **★** | **★** | **★★** | **★** |
| **Outcome** |  |  |  |  |  |  |  |  |  |  |
| Assessment of outcome | **★** | **★** | **★** | **★** | **★** | **★** | **★** |  | **★** |  |
| Follow-up long enough for outcomes to occur | **★** | **★** | **★** | **★** | **★** | **★** | **★** | **★** | **★** | **★** |
| Adequacy of follow up of cohorts | **★** | **★** | **★** | **★** | **★** | **★** | **★** | **★** | **★** | **★** |
| **Total** | **9** | **7** | **9** | **7** | **8** | **9** | **7** | **7** | **9** | **7** |

**Table S2.** Detailed quality assessment of cohort studies.

A study can be awarded a maximum of one star for each numbered item within the Selection and Outcome categories. A maximum of two stars can be given for Comparability. Study rates ≥6 is eligible for further analysis. NOS, Newcastle-Ottawa Scale.
